# Elizabeth Fleischman-Aschheim: A Pioneer for X-Ray Safety

**DOI:** 10.7759/cureus.82330

**Published:** 2025-04-15

**Authors:** Ananya Surabhi, Latha Ganti

**Affiliations:** 1 Emergency Medicine, Brown University, Sugar Land, USA; 2 Medical Science, The Warren Alpert Medical School of Brown University, Providence, USA; 3 Research, Orlando College of Osteopathic Medicine, Winter Garden, USA; 4 Emergency Medicine & Neurology, University of Central Florida, Orlando, USA

**Keywords:** general radiology, historical vignette, history of medical sciences, personal protective equipment patient safety healthcare worker safety, radiology safety

## Abstract

Elizabeth Fleischmann-Aschheim (March 5, 1867, to August 3, 1905) was a trailblazing radiographer whose contributions to early X-ray technology reshaped medical diagnostics and set the foundation for modern radiographic safety. At a time when the medical community was still uncovering the possibilities of X-rays, she boldly stepped into an uncharted field, demonstrating both technical expertise and determination to advance scientific knowledge regardless of the risk. Despite facing societal and economic challenges, Fleischmann’s unwavering dedication to her craft not only revolutionized radiography but also emphasized the urgent need for safety measures, an aspect tragically reinforced by her fate as the first woman to die of X-ray radiation exposure.

## Introduction and background

In 1895, Wilhelm Röntgen discovered X-rays [1-3]. By 1896, a year after X-rays were first invented, scientists across the world were investigating their uses [1,4]; researchers reported using X-rays to improve skin lesions, eradicate cancer, and work as an agent against bacterial infection [1,4]. Some artists even began to explore the field as a new creative medium [5-7]. Thus, Roentgen was awarded the first Nobel Prize for Physics in 1901 because he uncovered this versatile technology [3,4].

In the years following this exciting discovery, little attention was immediately given to the possible danger X-rays posed. The early use of X-rays was primarily unrestricted and versatile [3]. Elizabeth Fleischmann-Aschheim was among the scientific community who were enthralled with the scientific advancement and experimented with X-rays frequently, on herself and patients, while learning about the field. Her contributions to the field of X-ray technology advanced the ability of practitioners to use it safely. Her success was achieved despite economic and societal challenges.

## Review

Early life and entry into radiography

**Figure 1:**
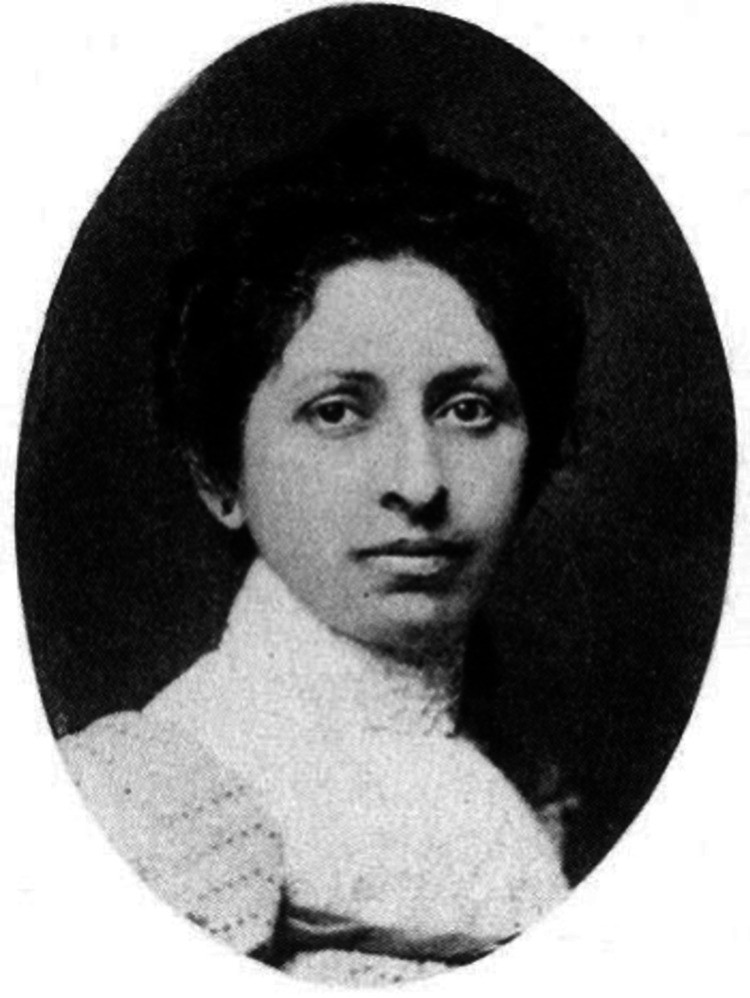
Portrait of Elizabeth Fleischmann-Aschheim From: https://www.foundsf.org/index.php?title=X-ray_Photographer:_Elizabeth_Fleischmann Content is available under Creative Commons Attribution-Noncommercial-Share Alike 3.0. Powered by MediaWiki

Born in 1867 in El Dorado County, California, to Austrian Jewish immigrants, Elizabeth Fleischmann-Aschheim grew up in modest circumstances [5,8-10]. Financial difficulties forced her to leave high school early, redirecting her focus toward supporting her family [5,8,10]. However, her intellectual curiosity never waned. Thus, when possible, she took courses in accounting and business to allow her to advance and better support herself and her family [8]. In her early life, she worked as a baker and a merchant [5,8] (Figure 1).

**Figure 2:**
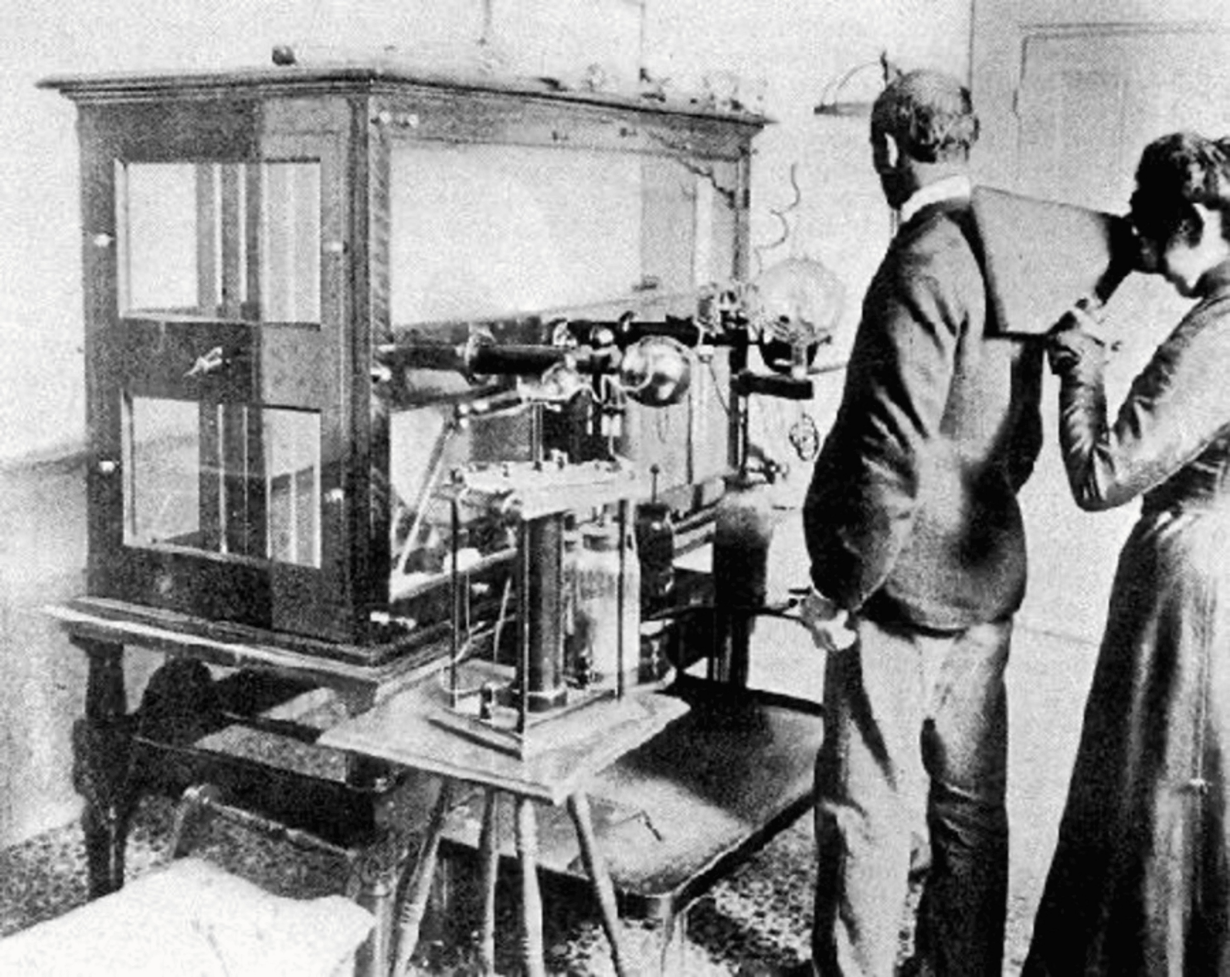
Elizabeth Fleischmann-Aschheim using a portable fluoroscope prior to her death in 1905 From: https://www.foundsf.org/index.php?title=X-ray_Photographer:_Elizabeth_Fleischmann Content is available under Creative Commons Attribution-Noncommercial-Share Alike 3.0. Powered by MediaWiki

When Fleischmann’s mother died, she moved in with her sister, Estelle, and brother-in-law, Dr. Michael J.H. Woolf, a San Francisco physician [8-9,11]. She worked as a bookkeeper for Dr. Woolf, who was the one to introduce Fleischmann to X-ray technology [8-9,11]. Her interest in the emerging field of radiography was life-changing. She read all available materials and attended a public lecture by Albert Van der Naillen, who later accepted Fleischmann to his School of Engineering, where she studied electrical science [9]. She borrowed money to purchase an X-ray machine and fluoroscope that was delivered and stored in Dr. Woolf’s office, which likely means it was the first privately owned apparatus in California [5 8-9,11] (Figure 2).

She remained a single, Jewish entrepreneur until her mid-30s when she married Israel Julius Aschheim and uniquely chose to hyphenate her last name [5].

Determined to master the new technology, Fleischmann immersed herself in hands-on experimentation. She was able to practice with the newly available X-ray apparatus on chloroformed animals and human patients [8]. By mid-1896, she had established California’s first X-ray laboratory at 611 Sutter Street in San Francisco, becoming one of the earliest professional radiographers in the United States [5,8-9,11]. At a time when few women were involved in scientific pursuits, Fleischmann’s achievements were groundbreaking. She remained without formal education and titles behind her name, yet she was accepted into the scholarly radiography sphere. Her laboratory delivered images to local physicians and quickly became known for producing high-quality radiographs [5,8-9,11].

Contributions to radiography

Fleischmann’s work extended far beyond the confines of her laboratory. By 1898, the United States Army enlisted her help [12,13]. During the Spanish-American War, she used X-ray technology to locate bullets and shrapnel in wounded soldiers returning from the Philippines [12,13]. Her precise imaging skills provided life-saving assistance to military surgeons, earning her praise from the Surgeon General of the Army [8-9,11-13]. In 1900, her expertise was recognized as she became an inaugural member of the Roentgen Society of the United States [14]. She also served as the first woman and one of the few members from the United States of the society’s academic journal, The Archive of Roentgen Ray, the editorial committee [14]. As one of the few non-physician society members, Fleischmann’s inclusion underscored her extraordinary standing in the field of radiography. She not only mastered the art of producing X-ray images, but she also proved the immense value of the technology in medical diagnostics long before it became a standard practice.

The emerging field of radiography

At the dawn of radiography, the dangers of X-ray exposure were poorly understood [1,3-4]. Physicians, researchers, and radiographers often operated without protective measures, unaware of the long-term health risks [1,3-4]. Like many other scientists, Fleischmann was initially distracted by the seemingly miraculous technology and did not consider that it was quietly causing damage with each exposure [8]. X-rays were being used to experiment on a number of “ailments,” from ringworm to tuberculosis to birthmarks [1,3-4]. Early experimenters like Thomas Edison and Nikola Tesla reported halting further work with X-rays and radioactive materials after experiencing eye and skin irritation [1,3]. However, communication and data sharing occurred at a much slower pace; thus, X-rays remained present in popular society and the scientific community [13].

Pioneering X-ray safety measures

Despite slower communication channels, it became evident fairly quickly that X-rays had significant associated hazards [1]. Elizabeth Fleischmann-Aschheim joined The Roentgen Society in 1900 as the inaugural member from the United States [14]. This organization established a committee “to report on the alleged injurious effects” of X-rays [1]. Despite being one of the few members of society who were not physicians, Fleischmann was among the first to recognize the potential hazards associated with prolonged radiation exposure. As early as 1904, she began advocating for protective measures, urging X-ray operators to use double-plate glass screens and experimenting with various shielding materials, including lead, aluminum, iron, and copper [15]. Unfortunately, the first professional radiation safety recommendations were not published until 1913 and only urged simple safe practices, such as staying as far as possible from the X-ray tube when energized and employing shielding (possibly lead) around the X-ray source [16].

Her forward-thinking approach marked a significant step toward the development of radiation safety protocols. Tragically, despite her own precautions, the cumulative effects of radiation exposure took a devastating toll on her health [5,8,9,11]. By 1905, she suffered severe radiation burns, which led to the amputation of her right arm [5,8,9,11]. The damage was irreversible. Later that year, Fleischmann succumbed to complications from radiation poisoning [5,8,9,11]. Her death was a somber testament to the occupational hazards faced by early radiographers, reinforcing the urgent need for stricter safety regulations.

## Conclusions

Elizabeth Fleischmann-Aschheim’s contributions to radiography and radiation safety continue to resonate within the medical community. Her pioneering work demonstrated the transformative power of X-rays in medical diagnostics, and her early warnings about radiation exposure paved the way for the development of modern safety standards. Even though her life was tragically cut short, her legacy endures. Today, radiologists and medical professionals benefit from the advancements in radiation protection that she helped to inspire. Fleischmann’s story serves as both a cautionary tale and a testament to the spirit of scientific discovery, a reminder of the sacrifices made by those who pushed the boundaries of knowledge to improve the lives of others. Her name may not be as widely recognized as some of her contemporaries, but Elizabeth Fleischmann remains a true pioneer in the field of radiography. Through her work, she not only illuminated the human body but also shed light on the necessity of safety in scientific innovation.
